# Ion‐Driven Regulation of Ca^2^^+^ Flow by Ti/Zn Composite Activates ERK/MAPK Signaling and Maintains Mitochondrial Homeostasis to Promote Macrophage‐Mediated Bone Regeneration

**DOI:** 10.1002/advs.76941

**Published:** 2026-08-05

**Authors:** Wanyi Huang, Qiyue Zhang, Yueyang Qiu, Haocheng Suo, Zengqian Liu, Bing Bai, Qiang Wang, Qing Zhou

**Affiliations:** ^1^ School and Hospital of Stomatology China Medical University Shenyang People's Republic of China; ^2^ Liaoning Provincial Key Laboratory of Oral Disease Shenyang People's Republic of China; ^3^ Shi‐Changxu Innovation Center for Advanced Materials Institute of Metal Research Chinese Academy of Sciences Shenyang People's Republic of China; ^4^ School of Materials Science and Engineering University of Science and Technology of China Hefei People's Republic of China

**Keywords:** biomaterials, macrophage polarization, mitochondrial homeostasis, osteogenesis, zinc

## Abstract

The field of tissue engineering is transitioning from bioinert to bioactive materials that actively regulate host biology. Here, we developed a 3D‐printed porous Ti scaffold infiltrated with molten Zn to form an interpenetrating Ti/Zn composite with immunomodulatory and osteogenic bioactivity. Corrosion characterization revealed sustained and stable Zn^2+^ release. In vivo evaluation using a rat rib implantation model confirmed enhanced peri‐implant bone regeneration, accompanied by localized Ca/P deposition at Zn degradation sites, indicating biofunctionally favorable degradation. In vitro, Ti/Zn extract induced a shift toward an anti‐inflammatory M2 macrophage phenotype. Mechanistically, Zn^2+^ altered intracellular Ca^2+^ dynamics, elevating cytoplasmic Ca^2+^ while preventing mitochondrial Ca^2+^ overload, thereby preserving mitochondrial membrane potential and inner membrane protein complex stability. This Ca^2+^ redistribution selectively activated ERK/MAPK signaling, linking ionic cues to mitochondrial homeostasis, reduced ROS generation, and suppression of cytochrome c‐mediated intrinsic apoptosis. The immunoregulatory secretome from Ti/Zn‐reprogrammed macrophages further promoted osteoblast proliferation, ALP activity, and osteogenic gene/protein expression. In vivo immunohistochemistry and immunofluorescence validated a remodeled immune microenvironment with attenuated oxidative stress and active new bone synthesis. Overall, the Ti/Zn composite integrates bone‐mimetic architecture with ion‐driven immunometabolic regulation, highlighting its potential as a next‐generation bioactive bone implant.

## Graphical abstract

A 3D‐printed interpenetrating Ti/Zn composite enables sustained Zn^2+^ release to regulate intracellular Ca^2+^ redistribution, preserve mitochondrial homeostasis, activate ERK/MAPK signaling, and promote M2 macrophage polarization, thereby enhancing osteoimmune‐mediated bone regeneration and peri‐implant osseointegration.

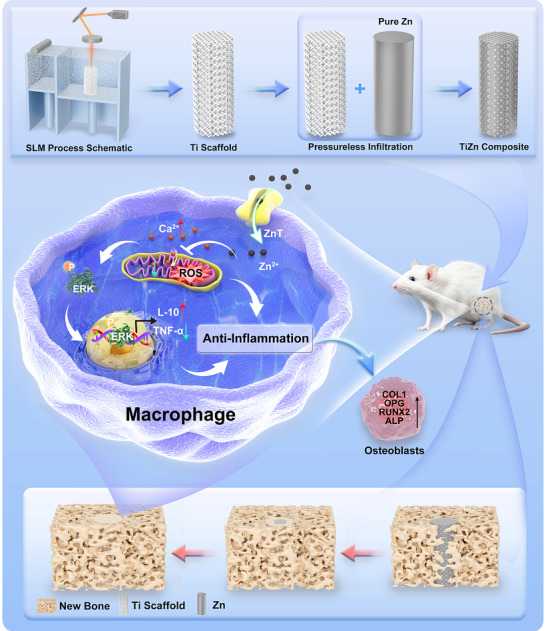

## 1 Introduction

The regeneration of bone tissue relies on the development of advanced materials and fabrication processes and remains a vital treatment strategy for bone defects [1]. Metallic biomaterials offer distinct advantages, such as excellent biocompatibility, strong mechanical strength, intrinsic bioactivity, and cost‐efficient manufacturing, making them especially promising for bone tissue engineering applications compared to other biomaterials, including polymers and ceramics [2]. Titanium (Ti) and its alloys are widely utilized in orthopedics and dentistry due to their excellent biocompatibility and mechanical properties [3]. However, their inherent bioinertness limits broader clinical applications [4]. This bioinert nature often triggers a foreign body reaction (FBR)—an acute sterile inflammatory response that can disrupt tissue vascularization and remodeling, potentially leading to fibrotic encapsulation. This encapsulation creates a barrier, hindering integration between the implant and surrounding tissues [5]. However, FBR is not an inevitable outcome of implantation. The key to successful integration lies in modulating the host immune response to promote tissue regeneration instead of encapsulation [6, 7]. Recognizing this, the field of tissue engineering is shifting away from bioinert materials toward bioactive matrices designed to actively guide beneficial host responses and improve integration [8, 9].

Zinc, an essential mineral vital for human growth, immune function, and tissue repair, plays a crucial role in skeletal development and bone homeostasis [10]. Distributed primarily in muscle (57%) and bone (29%), zinc is concentrated primarily at sites of tissue calcification, such as osteons and calcified cartilage [11]. Research has shown that zinc supplementation promotes bone formation and fortifies bone strength, while zinc deficiency can lead to bone fragility and compromised health [12, 13]. As a degradable metal, zinc exhibits a moderate degradation rate compared to other potential orthopedic degradable metals like magnesium and iron [2]. Furthermore, zinc does not produce hydrogen gas during the degradation process [14]. Therefore, zinc‐based biomaterials, with their favorable biodegradability, antibacterial properties, and capacity to promote angiogenesis and osteogenesis, are proving increasingly valuable in tissue engineering applications.

Although the structural and catalytic functions of zinc have long been recognized, its role in cell signaling has only recently come to light. Known as the “calcium” of the twenty‐first century, zinc is essential for numerous biological functions, including nucleic acid metabolism, stimulation of bone formation, signal transduction, bone mass maintenance, apoptosis regulation, and gene expression [15, 16]. Zinc homeostasis is a complex process governed by various receptors and involves multiple organelles [17]. Notably, zinc's effects vary based on cell type and biological context [18]. However, current research on zinc metabolism and intracellular mechanisms is concentrated mainly in fields such as neurobiology and oncology, with most studies on zinc's impact on the immune system focusing on adaptive immunity [19, 20, 21]. The signaling pathways of zinc within innate immune cells remain insufficiently understood.

Bone macrophages, alongside osteogenic lineage cells and osteoclasts, are now recognized as a third key cellular component in bone tissue [22]. They are involved in immunity, tissue repair, and tissue homeostasis [23]. Different phenotypes of macrophages interact with osteoclasts and osteoblasts at various levels to regulate normal bone homeostasis [24, 25]. M1 and M2 macrophages represent two primary functional types, each playing distinct roles in the immune response and tissue homeostasis. M1 macrophages are known as the “classically activated” macrophages and are associated with pro‐inflammatory responses. When activated by signals like interferon‐gamma (IFN‐γ) or microbial products (e.g., lipopolysaccharides), M1 macrophages produce high levels of inflammatory cytokines, reactive oxygen species (ROS), and nitric oxide (NO) [26]. These factors are essential for fighting infections and breaking down pathogens, making M1 macrophages critical for initiating the immune response. However, excessive M1 activity can lead to chronic inflammation and tissue damage [27]. M2 macrophages, on the other hand, are “alternatively activated” and are primarily associated with anti‐inflammatory functions and tissue repair. Stimulated by signals like interleukin‐4 (IL‐4) or interleukin‐13 (IL‐13), M2 macrophages produce anti‐inflammatory cytokines and growth factors, which help in wound healing, tissue remodeling, and resolving inflammation [28]. They are involved in clearing cellular debris and promoting regeneration, making them vital for restoring homeostasis after an immune response [29]. The balance between M1 and M2 macrophage polarization is essential in various physiological and pathological contexts, including inflammation, tissue repair, and regenerative medicine [30]. Macrophage‐targeted strategies in biomaterial applications play a crucial role in promoting bone regeneration and healing bone defects. These approaches are emerging as essential in addressing bone loss and repairing bone deficiencies. Current advancements highlight the importance of immunomodulatory biomaterials, with a primary focus on enhancing the polarization from M1 to M2 macrophages [31, 32].

Additive manufacturing of porous metal scaffolds holds immense potential for treating bone defects, especially in high load‐bearing scenarios [33]. This technology offers unparalleled customization in design and fabrication, while the inherent properties of metals provide the necessary mechanical strength and stability. The porous structure further promotes long‐term implant fixation by enabling bone ingrowth, leading to mechanical interlocking between the implant and bone tissue. Recognizing these advantages, we fabricated a novel Ti/Zn composite by infiltrating molten zinc into a 3D‐printed porous titanium scaffold that reproduces trabecular architecture. This approach leverages the benefits of porous metallic scaffolds and imparts desirable immune‐modulating properties to the bioinert titanium. Furthermore, the controlled degradation of zinc over time creates space for new bone growth, ultimately enhancing the interlocking between the implant and bone, leading to superior integration and long‐term stability. This study investigates the physical properties and physiological function of the Ti/Zn composite in osteogenesis, specifically focusing on its immunomodulatory effects and mechanism of action in macrophages.

## 2 Materials and Methods

### 2.1 Fabrication of the Ti/Zn Composite

The fabrication methods of the Ti scaffolds are similar to our previous Mg‐Ti composite [34]. Briefly, cylindrical titanium (Ti) scaffolds, measuring 11 mm in diameter and 31 mm in height, were fabricated using selective laser melting (SLM) with an AmPro SP100 SLM system (Arcam AB, Sweden). The titanium scaffolds were designed with two pore sizes, 1200 µm and 1500 µm. After fabrication, the scaffolds were subjected to a two‐step cleaning procedure: initially, a 1‐min ultrasonic treatment in a 40 wt% hydrofluoric acid solution at 50 W to eliminate unfused Ti powder, and subsequently, a rinse with ethanol. To fabricate the Ti/Zn composite, the scaffolds were infiltrated with pure zinc through a pressureless infiltration process conducted in a flowing argon atmosphere at 600 °C for 30 min, followed by furnace cooling. The composite with a pore size of 1200 µm was denoted as Ti/Zn‐1200, while that with a pore size of 1500 µm was denoted as Ti/Zn‐1500. Samples for in vitro studies were prepared with dimensions of 10 mm diameter × 2 mm thickness, while those for in vivo studies were prepared with dimensions of 1.2 mm diameter × 10 mm length. All samples were polished with silicon carbide (SiC) papers to a grit of 800–2000 and rinsed in distilled water for 10 min. The samples underwent sequential ultrasonic etching in acetone and ethanol before sterilization under ultraviolet light for 40–60 min.

### 2.2 Calculating Scaffold Porosity and Zinc Volume Proportion in Ti‐Zn Composite

Porosity values for the titanium scaffolds are calculated according to the following equation:

$$\mathrm{Porosit}y_{\mathrm{Ti}}=\left(1-\frac{\rho_{\mathrm{Ti}}}{\rho_{0}}\right)$$

where ρ_0_ is non‐porous Ti6Al4V(4.43 g/cm^3^), and ρ_Ti_ is the density of the manufactured scaffolds, calculated using the weight and volume of the scaffolds.

$$\mathrm{Zn}\varphi B=\left(1-\frac{\rho_{\mathrm{Ti}}}{\rho_{0}}\right)$$

### 2.3 Microstructural Characterization

Microstructural characterization was performed using a LEO Supra‐35 field‐emission scanning electron microscope (SEM) (LEO, Germany) operating at 20 kV. Elemental analysis was conducted via energy‐dispersive X‐ray spectroscopy (EDS) using an Oxford Model 7426 spectrometer (Oxford Instruments, UK) attached to the SEM. The phase composition was determined by X‐ray diffraction (XRD) using a Bruker D8 Advance diffractometer (Bruker AXS, Germany) with Cu‐Kα radiation.

### 2.4 Immersion Test

For the immersion test, samples were placed in 24‐well cell culture plates and immersed in Dulbecco's Modified Eagle Medium (DMEM) at 37 °C, maintaining an immersion ratio of 1.25 cm^2^/mL. The DMEM was refreshed every 24 h, and the pH was measured at regular intervals using a PHS‐3C pH meter (Leici, China). An 8 mL aliquot was collected and filtered at each designated time point through a 0.22 µm Millex‐GS filter (Millipore Corp., Bedford, MA). The concentration of Zn^2+^ ions in the filtrate was determined using an inductively coupled plasma mass spectrometer (YQ‐192, Shimadzu, Kyoto, Japan). Zn^2+^ release studies were performed in triplicate, and the average Zn^2+^ concentration was calculated for each time point. Following immersion, corrosion products were removed with Kroll's Reagent for 5 s and dried with hot air. According to ASTM‐G31‐72, CR was deduced from the tests by the following equation:

CR=KW/ATρ

where constant *K* = 8.76 × 10^4^, *W* is mass loss accurate to 1 mg, *A* is the total immersed surface accurate to 0.01 cm^2^, *T* is the immersion time with a unit of h, *ρ* is sample density with a unit of g/cm^3^. Sample morphology was documented using a digital camera, and the microstructure was analyzed via scanning electron microscopy (SEM) and energy‐dispersive X‐ray spectroscopy (EDS). Nanoindentation testing was used to evaluate the mechanical properties of the Ti/Zn composite during immersion, and the measurements were conducted using a nanoindentation tester (Agilent G200, USA).

### 2.5 In Vivo Experiments

#### 2.5.1 Rat Model

The Ethics Committee of China Medical University approved all animal surgical procedures in the study (CMU20240587). Eight‐week‐old male Sprague Dawley rats (200–250 g, Changsheng Biotechnology, China) were used in this study. Rats were anesthetized using isoflurane (3% induction, 2% maintenance) delivered via an anesthesia mask, with continuous monitoring of anesthesia depth. After shaving and immobilizing the hind limb in the supine position, a 5 mm longitudinal incision was made to expose the distal femur. A cylindrical hole (1.2 mm diameter, 10 mm length) was created along the femoral axis, and either a Ti implant (left leg) or a Ti/Zn‐1500 implant (right leg) was inserted. Following implantation, soft tissues were closed, and the skin was sutured. Post‐operatively, rats received 200 µL of penicillin (200,000 units/mL) injections and topical erythromycin for 3 days to prevent infection. At predetermined time points, rats were euthanized via CO_2_ asphyxiation. Bilateral femurs were harvested and fixed in 4% paraformaldehyde.

#### 2.5.2 Micro‐CT Analysis

Harvested rat femurs were scanned using a microcomputed tomography (micro‐CT) scanner (Zeiss Xradia 520 Versa, Germany) at 45 kV and 200 mA. Three‐dimensional (3D) images were reconstructed using CTvox 3.0 software (Bruker, Germany). A cylindrical region of interest (ROI) was defined using CTAn (version 1.15.2.2, Bruker, Germany), with a diameter of 0.3 mm around the implant and a length of 4 mm extending into the medullary cavity. Within the ROI, histomorphometric indices, including bone volume fraction (BV/TV), trabecular number (Tb.N), trabecular separation (Tb.Sp), and trabecular thickness (Tb.Th), were quantitatively analyzed.

#### 2.5.3 Histological Staining

Rat femurs were processed for hard tissue slicing and staining. Briefly, femurs were rinsed in water, dehydrated in a graded ethanol series, cleared in xylene, and embedded in methyl methacrylate. Longitudinal sections (3–4 per femur, 200 µm thick) were cut parallel to the implant axis and ground down to 100 µm. Sections were stained with a Masson stain kit (Beyotime, China) and a TRAP stain kit (Beyotime, China). SEM and EDS were conducted on hard‐tissue sections to assess the in‐tissue degradation of the Ti/Zn composite and the associated new bone regeneration.

To assess biosafety, vital organs (heart, liver, spleen, lung, and kidney) were harvested at 8 weeks post‐implantation, fixed in 10% neutral buffered formalin, and processed for histological examination. Briefly, organs were dehydrated, embedded in paraffin, sectioned, and stained with hematoxylin and eosin (H&E) to evaluate potential pathological changes.

Immunohistochemistry (IHC) staining and immunostaining were performed to identify macrophage polarization. Femurs were decalcified in 10% ethylenediaminetetraacetic acid (EDTA) for 8 weeks, dehydrated in a graded ethanol series, embedded in paraffin, and sectioned. Sections were dewaxed, rehydrated, and subjected to antigen retrieval. Sections were then incubated overnight at 4 °C with primary antibodies against TNF‐α (Abcam, UK), IL‐10 (Abcam, UK), CD86 (Abcam, UK), RUNX‐2 (1:1000, Invitrogen, USA), OCN (Invitrogen, USA) followed by incubation with corresponding secondary antibodies for 30 min. The histological images were captured using a digital scanner (Shengqiang, China) and confocal microscope (Nikon, Japan). Images are analyzed using Image J.

### 2.6 In Vitro Experiments

#### 2.6.1 Extract Preparation

Samples were immersed in DMEM supplemented with 10% fetal bovine serum (FBS) and incubated at 37 °C in a humidified atmosphere of 5% CO_2_ for 72 h. The immersion ratio followed the ISO 10993 standard (1.25 cm^2^/mL, as previously mentioned). Subsequently, the extracts were filtered with a 0.22 µm filter and diluted fivefold for in vitro testing.

#### 2.6.2 Cell Cultures

RAW 264.7 cells were cultured in DMEM supplemented with 10% fetal bovine serum (FBS, Clark, USA) and 1% penicillin/streptomycin (Gibco, USA). MC3T3‐E1 cells were cultured in Alpha Minimum Essential Medium (α‐MEM) supplemented with 10% FBS (Clark, USA) and 1% penicillin/streptomycin (Gibco, USA). Both cell types were maintained at 37 °C in a humidified atmosphere of 5% CO_2_. For osteogenic differentiation, MC3T3‐E1 cells were cultured in α‐MEM supplemented with 10 mM β‐glycerophosphate disodium, 50 µg/mL ascorbic acid, and 100 nM dexamethasone. Cells were passaged at 80–90% confluence. RAW264.7 and MC3T3‐E1 cells were obtained from the Central Laboratory of School and Hospital of Stomatology affiliated with China Medical University.

#### 2.6.3 Reagents

TPEN, a high‐affinity zinc chelator, and PD98059, an ERK1/2 signaling inhibitor, were purchased from MedChem Express (New Jersey, USA). Flou‐3 and Rhod‐2 were purchased from Thermo Fisher Scientific (USA). JC‐1 analyze kit was purchased from Beyotime (China). Actin‐Tracker Green‐488 was purchased from Beyotime (China). LysoTracker Red DND‐99 was purchased from Yeasen (China).

#### 2.6.4 CCK‐8 Assay

The effects of Ti/Zn and pure Ti extracts on cell viability were evaluated using the Cell‐Counting Kit‐8 (CCK‐8) assay, following the manufacturer's instructions. For cytotoxicity assessment, RAW 264.7 cells were seeded in 96‐well plates (3 × 10^3^ cells/well) and incubated for 24 h. After washing with PBS, the cells were treated with 100 µL of either material extracts or fresh culture medium. At specific time points, the supernatant was discarded, and 100 µL of DMEM containing 10% CCK‐8 reagent was added to each well. Following a 2‐h incubation, absorbance was measured at 450 nm using a microplate reader (Infinite M200, Tecan, Australia).

To assess cell proliferation, MC3T3‐E1 cells were seeded (10^3^ cells/well) and incubated for 24 h before being washed and treated with 100 µL of either conditioned medium or fresh culture medium. At specific time points, the supernatant was discarded, and 100 µL of α‐MEM containing 10% CCK‐8 reagent was added. After a 2‐h incubation, absorbance was measured at 450 nm.

#### 2.6.5 Sequence

RAW264.7 cells were treated with Ti/Zn composite extract, Ti extract, or control medium (*n* = 3) for 6 h. Total RNA was isolated using TRIzol reagent (Takara, Japan) for RNA sequencing. For metabolomics profiling, cells were collected and centrifuged to remove the supernatant before sample preparation. RNA sequencing, metabolomics analysis, and bioinformatic processing were carried out by GeneChem (Shanghai, China).

#### 2.6.6 ELISA

RAW264.7 cells were seeded in triplicate wells of 6‐well plates at a density of 1 × 10^5^ cells/well and incubated overnight. Cells were then treated with extracts for 6 h. Subsequently, the supernatant was collected and centrifuged at 2000 rpm for 20 min at 4 °C. The concentrations of TNF‐α and IL‐10 in the supernatant were quantified using enzyme‐linked immunosorbent assay (ELISA) kits (Boster, China) according to the manufacturer's instructions.

#### 2.6.7 Flow Cytometry

RAW264.7 cells were seeded in triplicate wells of six‐well culture plates at a density of 1 × 10^5^ cells/well and incubated for 6 h. Cells were then collected, washed thrice with phosphate‐buffered saline (PBS), and stained with allophycocyanin (APC)‐conjugated CD86 (Invitrogen, USA) for 30 min. After two additional PBS washes, cells were resuspended in 300 µL of PBS and analyzed using a BD flow cytometer (BD, USA). Data analysis was performed using FlowJo software.

#### 2.6.8 PCR

RAW264.7 and MC3T3‐E1 were collected at various time points for RNA isolation. Total RNA was extracted using TRIzol reagent (Takara, Japan), and cDNA was synthesized using the HiScript II Q RT SuperMix for qPCR (Vazyme, China). Quantitative PCR (qPCR) was performed in duplicate using a ChamQ Universal SYBR qPCR Master Mix (Vazyme, China) on a 7500 Real‐Time PCR System (Applied Biosystems, USA). Primer sequences are available upon request. Target gene expression was normalized to the endogenous control gene, Actin, using the 2^–ΔΔCt^ method. The primer sequences are listed in Table S1. The specificity of the qPCR primers was verified and is presented in Figure S1. Data are presented as mean ± standard error of the mean (SEM) from three independent experiments.

#### 2.6.9 Western Blot

Protein was extracted from samples using radioimmunoprecipitation assay (RIPA) buffer (Beyotime) at 4 °C for 30 min. Lysates were centrifuged at 21,100 × *g* for 25 min at 4 °C, and the supernatant was collected. Protein concentration was determined using a BCA assay kit (Beyotime). Protein samples were separated by SDS‐PAGE using 12% gels and transferred to nitrocellulose membranes. After blocking, membranes were incubated overnight at 4 °C with gentle agitation with primary antibodies against the following targets: p‐ERK (1:1000, Servicebio, China), ERK (1:1000, Servicebio, China), p‐p38 (1:1000, Santa Cruz Biotechnology, USA), p38 (1:1000, Santa Cruz Biotechnology, USA), p‐cJUN (1:1000, Cell Signaling, USA), cJUN (1:1000, Cell Signaling, USA), Scl30A3 (1:1000, Proteintech, China), COL1 (1:1000, Abcam, USA), OPG (1:1000, Abcam, USA), RUNX‐2 (1:1000, Invitrogen, USA), ALP (1:1000, Invitrogen, USA), Cytochrome‐c (1:1000, Proteintech, China), Bcl‐1 (1:1000, Proteintech, China), MCU (1:200, Proteintech, China). Membranes were then washed three times with PBS and incubated for 1 h with HRP‐conjugated secondary antibodies (anti‐rabbit or anti‐mouse, 1:2000, R&D, USA). Finally, protein bands were visualized by an enhanced chemiluminescence detection system (ECL kit; GE Healthcare).

#### 2.6.10 Immunostaining

RAW264.7 cells were seeded onto sterile glass coverslips placed in six‐well culture plates at a density of 1 × 10^5^ cells/well and incubated overnight. MC3T3‐E1 cells were seeded onto sterile glass coverslips placed in 24‐well plates at a density of 2 × 10^4^ cells/well and incubated overnight. Cells were then treated with extracts for 6 h and fixed by 4% paraformaldehyde. The staining procedures were followed the user manual. The images were captured using confocal microscope (Nikon, Japan). Images are analyzed using Image J.

#### 2.6.11 Alkaline Phosphatase (ALP) Activity

For ALP staining, MC3T3‐E1 cells were seeded in 24‐well plates at a density of 2 × 10^4^ cells/well. After 24 h, the culture medium was replaced with conditioned medium, and the cells were incubated for 7 days. Following incubation, cells were washed once with PBS and fixed with 4% paraformaldehyde for 1 h. After two additional PBS washes, cells were stained using a 5‐bromo‐4‐chloro‐3‐indolyl phosphate/nitro blue tetrazolium (BCIP/NBT) alkaline phosphatase (ALP) color development kit (Beyotime, China), according to the manufacturer's instructions. Images of three randomly selected fields per well were captured using an inverted microscope (Nikon Ts2R, Japan). ALP staining was quantified using Image J software.

For quantitative analysis of alkaline phosphatase (ALP) activity, MC3T3‐E1 cells were seeded in triplicate wells of six‐well plates at a density of 1 × 10^5^ cells/well. After 24 h, the culture medium was replaced with conditioned medium. After 7 days of incubation, ALP activity was measured using an Alkaline Phosphatase Assay Kit (Beyotime, China) according to the manufacturer's instructions. Protein concentration was determined using a BCA protein assay kit (Beyotime, China). ALP activity was normalized to the total protein content for each sample.

#### 2.6.12 Alizarin Red S (ARS) Staining

For ARS staining, MC3T3‐E1 cells were seeded in 6‐well plates at a density of 5 × 10^5^ cells/well, co‐cultured with CTLCM or TiZnCM for 28 days, and were stained by Alizarin Red S Solution (OriCell, China). Stained cells were imaged under a light microscope.

### 2.7 Statistical Analysis

Results are presented as mean ± standard error of the mean (SEM). Statistical analysis was performed using SPSS 20 software. Data were analyzed using one‐way analysis of variance (ANOVA) followed by *t*‐test. Differences were considered statistically significant at *p* < 0.05.

## 3 Results

### 3.1 Microstructure of Ti/Zn Composite

Figure 1A shows the overall appearance of the 3D‐printed porous Ti scaffolds and the Ti/Zn composites after zinc infiltration. The composite with a pore size of 1200 µm was denoted as Ti/Zn‐1200, while that with a pore size of 1500 µm was denoted as Ti/Zn‐1500. The porosity of both the scaffold and the composite was calculated using a geometric method in Table S2, confirming successful zinc infiltration and composite formation. Scanning electron microscopy (SEM) coupled with energy‐dispersive X‐ray spectroscopy (EDS) was employed to analyze the microstructure and elemental composition. SEM images reveal a smooth surface morphology without noticeable defects like pores or micro‐cracks, indicating a highly dense composite structure with interpenetrating Ti and Zn phases (Figure 1B). EDS analysis confirmed that titanium and zinc are the only elements present in the composite (Table S3). Linear EDS measurements across the interface reveal distinct regions of elemental distribution (Figure 1C). Region I, corresponding to the titanium scaffold, exhibits high Ti signal intensity. A distinct titanium–zinc mixed interface (Region II) is observed between the titanium and zinc phases, characterized by a consistent Ti/Zn ratio. Region III, corresponding to the infiltrated zinc, shows high Zn signal intensity.

**FIGURE 1:**
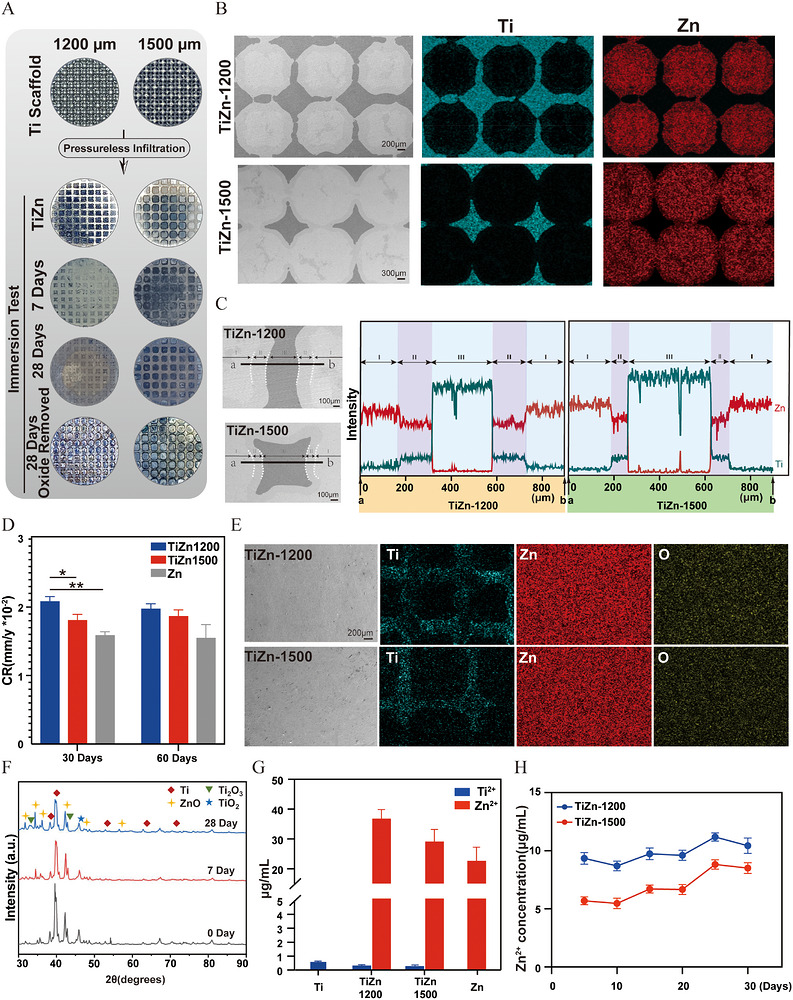
Morphology and corrosion behavior of the Ti/Zn composite. Ti/Zn‐1200 and Ti/Zn‐1500 refer to Ti scaffolds with pore sizes of 1200 µm and 1500 µm, respectively, following Zn infiltration. (A) Phtographic images of porous Ti scaffolds, corresponding Zn‐infiltrated composites and corroded Ti/Zn composite. (B) SEM images and EDS elemental maps of Ti/Zn composites. (C) Cross‐sectional SEM images and linear distribution of the elemental Zn and Ti through the interface between constituents. (D) The corrosion rate (CR) of the Ti/Zn composite and pure Zn in NS. (*n* = 5, * represents *p *< 0.05, ** represents *p *< 0.01, *t*‐test). (E) SEM image and corresponding EDS analysis of the Ti/Zn composite after 28 days of degradation. (F) XRD patterns of Ti/Zn‐1500 composites before and after immersion for different durations. (G) The concentration of Ti^2+^ and Zn^2+^ in the Ti/Zn composite, pure Ti and pure Zn extract. (*n* = 3). (H) The concentration of Zn^2+^ in the Ti/Zn composite extraction solution during daily fluid replacement, simulating body fluid circulation. (*n* = 3).

### 3.2 Corrosion Behavior of Ti/Zn Composite

The morphological changes of the Ti/Zn composite degradation over time are shown in Figure 1A. A thin layer of corrosion products formed on the Ti/Zn composite after one week of in vitro degradation, gradually thickening with extended immersion time. Following the removal of this oxide layer, zinc degradation becomes apparent, with the most significant zinc loss occurring at the Ti–Zn interface. Corrosion rates after 30 and 60 days were calculated based on weight loss following removal of the oxide layer (Figure 1D). Among the samples, Ti/Zn‐1200 exhibited the fastest degradation, followed by Ti/Zn‐1500 and then pure Zn. The corrosion layer on the immersed Ti/Zn composites was further examined by SEM and EDS (Figure 1E). Elemental analysis of this film reveals Ti, Zn, and O as the primary constituents, with zinc being the most abundant element, followed by oxygen. The XRD patterns of the Ti/Zn composite reveal its phase composition (Figure 1F, Figure S2), demonstrating that the oxide layer consists of Ti_2_O_3_, TiO_2_, and ZnO. Ion‐release analysis revealed a higher concentration of Zn^2+^ released from Ti/Zn‐1200 compared with Ti/Zn‐1500 and pure Zn (Figure 1G), which corresponded to the corrosion rate measurements (Figure 1D). In contrast, Ti^2+^ levels in the composite extracts were lower than those released from pure Ti, indicating that the presence of Zn provides a protective effect on Ti, likely due to preferential galvanic corrosion of Zn. To simulate physiological circulation, extraction medium was replaced daily, and the Zn^2+^ concentration was monitored. The results demonstrated a continuous and stable release of Zn^2+^ from the Ti/Zn composite (Figure 1H). These findings indicate that electrochemical corrosion occurs between Ti and Zn within the composite, accelerating the degradation of the Zn phase. Notably, the Ti/Zn‐1200 composite exhibited a faster degradation rate than the Ti/Zn‐1500 composite.

### 3.3 Mechanical Property Evolution of the Ti/Zn Composite during Immersion Testing

Nanoindentation testing was performed to evaluate the mechanical properties of the Ti/Zn composites and to monitor their evolution during immersion. For the Ti/Zn‐1200 composite, the Ti phase exhibited a marked increase in indentation displacement after 7 days of immersion, whereas the change in displacement between 7 and 28 days became much less pronounced (Figure 2A). In contrast, the Zn phase showed substantial displacement changes in both the 0–7 day and 7–28 day intervals, indicating continuous degradation‐related softening throughout the immersion period (Figure 2B). Correspondingly, the hardness of the Ti phase decreased progressively with immersion time (Figure 2C), while the Zn phase displayed a rapid hardness reduction during the first 7 days, followed by a slower decline from day 7 to day 28 (Figure 2D). For the Ti/Zn‐1500 composite, the Ti phase showed only a slight displacement increase after 7 days of immersion, and the displacement remained nearly unchanged from 7 to 28 days, although the 7‐day curve was steeper than that at 28 days (Figure 2E). The Zn phase in Ti/Zn‐1500 composite exhibited a relatively uniform and gradual change in load‐displacement behavior over the entire 0–28 day period (Figure 2F). Accordingly, the hardness of both the Ti and Zn phases in Ti/Zn‐1500 decreased steadily but moderately over the entire immersion period. Compared with Ti/Zn‐1200, the Ti/Zn‐1500 composite showed smaller displacement changes in both phases, indicating greater mechanical stability and slower degradation during immersion. Notably, the hardness of the Ti phase was approximately 5 GPa, closely matching that of natural bone, suggesting that this composite is particularly suitable for bone implant applications.

**FIGURE 2:**
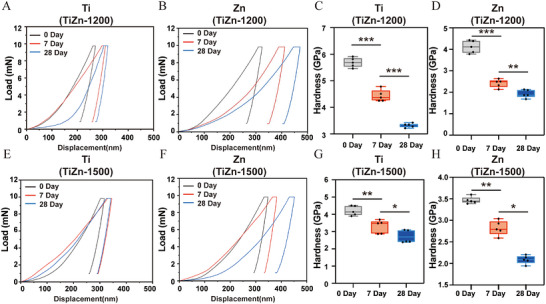
Mechanistic changes of Ti/Zn composite during immersion. Ti/Zn‐1200 and Ti/Zn‐1500 refer to Ti scaffolds with pore sizes of 1200 µm and 1500 µm, respectively, following Zn infiltration. (A, B) Nanoindentation load‐depth curves for Ti (A) and Zn (B) in Ti/Zn‐1200 composite. (C, D) Nanoindentation hardness in Ti (C) and Zn (D) in Ti/Zn‐1200 composite. (E, F) Nanoindentation load‐depth curves for Ti (E) and Zn (F) in Ti/Zn‐1500 composite. (G, H) Nanoindentation hardness in Ti (G) and Zn (H) in Ti/Zn‐1200 composite. (*n* = 5, * represents *p* < 0.05, ** represents *p* < 0.01, *** represents *p* < 0.001, *t*‐test).

In conclusion, the Ti/Zn‐1500 composite exhibited greater mechanical stability and slower degradation compared with Ti/Zn‐1200, while maintaining a Ti‐phase hardness (∼5 GPa) close to that of natural bone, highlighting its potential suitability for bone implant applications. Therefore, Ti/Zn‐1500 was selected for subsequent biological performance evaluations.

### 3.4 Ti/Zn Composite Promote Bone Regeneration in Rats

To investigate the in vivo degradation behavior of the Ti/Zn composite and its effect on peri‐implant osteogenesis, a rat femoral critical‐sized defect model was established. Rats were sacrificed at 1, 4, 8, and 12 weeks post‐implantation (Figure 3A). Micro‐CT analysis revealed continuous new bone deposition around the Ti/Zn implants (Figure 3B). Quantitative metrics demonstrated that the Ti/Zn composite significantly increased bone volume at week 1 and enhanced trabecular number by week 4 relative to pure Ti (Figure 3C), indicating accelerated early‐stage bone formation. This early osteogenic response is consistent with the biological roles of Zn ions, which are known to promote osteoblast differentiation, matrix maturation, and mineralization [35]. The close apposition of newly formed bone tissue to the composite surface supports the concept that the rigid Ti scaffold provides mechanical stability while the controlled release of Zn^2+^ contributes essential osteoactive cues in the peri‐implant microenvironment. To elucidate the degradation mechanism, SEM and EDS were used to evaluate the bone–implant interface at the microscale (Figure 3D–F). The Zn component exhibited corrosion features from week 4, which became more pronounced at week 8 and 12. Degradation products dispersed into the surrounding tissues. Elemental mapping showed localized Zn enrichment near the implant, spatially colocalized with regions of active new bone formation. TRAP staining demonstrated a reduced number of osteoclasts around the Ti/Zn composite, indicating suppressed bone resorption (Figure 3G). Furthermore, Masson's trichrome staining confirmed direct osteoid deposition around the Ti/Zn composite, suggesting enhanced bone‐implant integration (Figure 3H). These results suggest that Zn^2+^ is confined and sustained, creating a bioactive niche known to activate osteogenic signaling pathways (e.g., Runx2, ALP, collagen synthesis), without causing systemic overload. HE staining of major organs revealed no structural abnormalities, confirming that degradation products from the Ti/Zn composite were well tolerated (Figure S3). This systemic safety profile aligns with the controlled degradation rate and the physiologically acceptable range of Zn accumulation.

**FIGURE 3:**
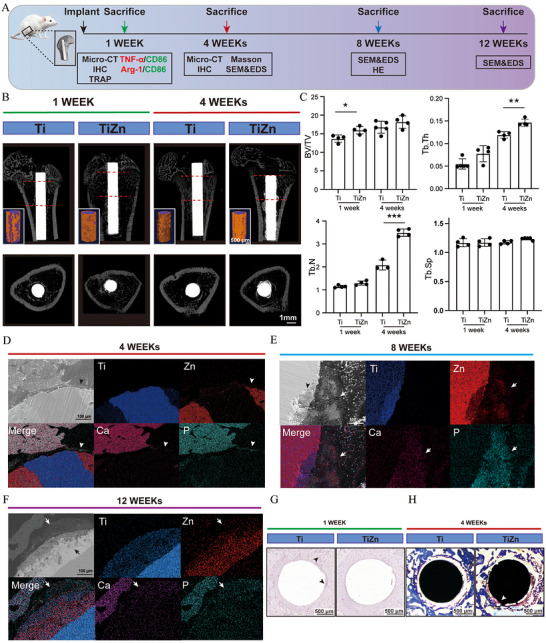
In vivo evaluation of osteogenesis induced by Ti/Zn composite. Ti/Zn represents the Ti/Zn‐1500 composite, which is a Zn‐infiltrated Ti scaffold with a pore size of 1500 µm. (A) Schematic representation of the experimental design. (B) Representative Micro‐CT image and 3D models derived from representative Micro‐CT scans incorporating bone tissue and the implant. (C) Quantitative analysis of the newly formed bone volume (BV/TV), the trabecular thickness (Tb.Th), the trabecular number (Tb.N), the trabecular separation/spacing (Tb.Sp). (* represents *p* < 0.05, ** represents *p* < 0.01, ***represents *p* < 0.001, *t*‐test). (D–F) Representative bone–implant interface and corresponding elemental mappings in the rat femoral critical‐sized defect model at 4 (D), 8 (E), and 12 (F) weeks (The arrow indicates the degradation of the zinc part of the Ti/Zn composite and the new bone formation.). (G) TRAP staining of the tissue around the implants (The arrow indicates the osteoclast accumulation.). (H) Masson staining of tissue around the implants (The arrow indicates the new bone formation.).

In conclusion, these findings demonstrate that the Ti/Zn composite undergoes predictable and spatially confined degradation in vivo while simultaneously releasing osteoactive Zn^2+^. The combination of Ti's mechanical integrity and Zn's biological functionality synergistically enhances peri‐implant bone regeneration. This dual‐mode action positions the Ti/Zn composite as a promising candidate for bioactive implant materials designed to modulate the local microenvironment and improve osseointegration.

### 3.5 Ti/Zn Composite Modulates the Immune Microenvironment in Peri‐Implant Tissue

To investigate whether the Ti/Zn composite regulates the immune microenvironment during early osseointegration, we first examined the expression of two key inflammatory cytokines, TNF‐α and IL‐10 (Figure 4A). The Ti/Zn composite markedly reduced TNF‐α expression while simultaneously enhancing IL‐10 levels in the peri‐implant tissue, indicating that local zinc release drives a transition from a pro‐inflammatory toward an anti‐inflammatory milieu. Such a shift is widely recognized as essential for suppressing chronic inflammation and supporting subsequent bone regeneration. To further elucidate the cellular basis of this immune modulation, dual immunofluorescence staining was performed to characterize macrophage phenotypes around the implants (Figure 4B–E). Macrophages were primarily localized along blood vessels and at the implant–tissue interface, regions typically involved in early immune activation and tissue remodeling. Notably, strong TNF‐α (M1‐associated) signals and Arg‐1 (M2‐associated) signals were both detected in these regions, but with distinct distribution patterns suggestive of phenotype transition. Compared with pure Ti, Ti/Zn implants showed attenuated M1‐associated TNF‐α signals and enriched Arg‐1 expression, confirming that zinc incorporation promotes a macrophage polarization shift from M1 to M2.

**FIGURE 4:**
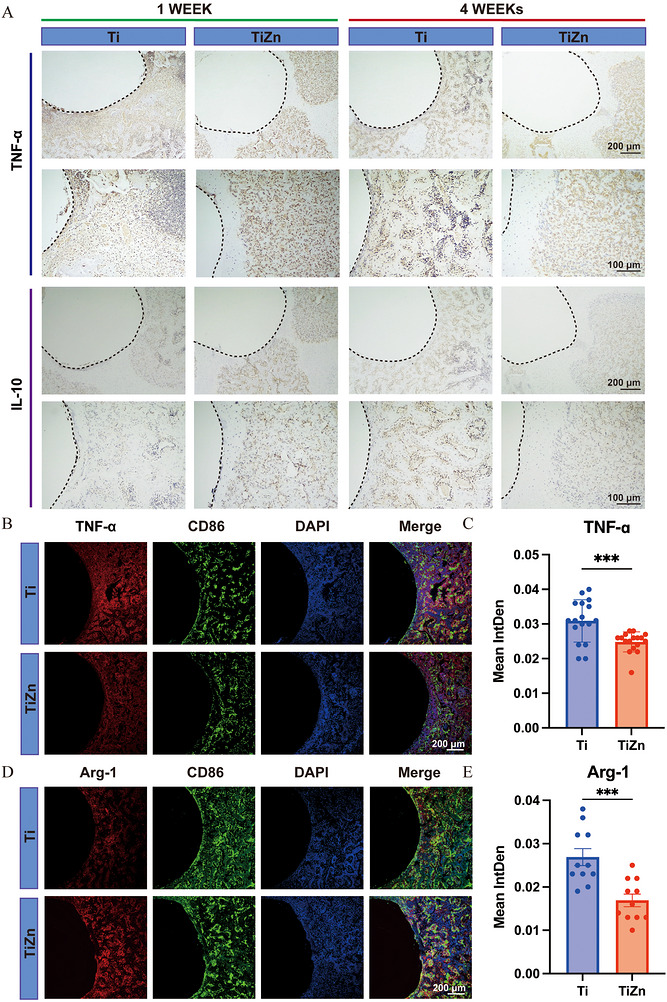
In vivo evaluation of immune microenviroment modulated by Ti/Zn composite. Ti/Zn represents the Ti/Zn‐1500 composite, which is a Zn‐infiltrated Ti scaffold with a pore size of 1500 µm. (A) Representative immunohistochemical images of TNF‐α and IL‐10 in peri‐implant tissue. (B) Dual immunofluorescence images of TNF‐α (red) and CD86 (green), illustrating macrophage responses regulated by the Ti/Zn composite. (C) Quantification analysis of (B). (D) Dual immunofluorescence images of IL‐10 (red) and CD86 (green), illustrating macrophage responses regulated by the Ti/Zn composite. (E) Quantification analysis of (D). (*n* = 10, *** represents *p* < 0.001, *t*‐test).

### 3.6 Ti/Zn Composite Induces M2 Macrophage Polarization In Vitro

To further corroborate the in vivo findings and dissect the immunomodulatory mechanisms of the Ti/Zn composite, the RAW 264.7 macrophage cell line was employed to evaluate macrophage polarization in vitro. Gene expression analysis revealed that cells cultured with the Ti/Zn composite exhibited upregulated M2‐associated markers, including Arg‐1, BMP‐2, and IL‐10 (Figure 5A–C), whereas M1‐associated genes, such as IL‐1β, iNOS, and TNF‐α, were significantly downregulated compared with Ti or control groups (Figure 5D–F). These transcriptional changes indicate that the Ti/Zn composite promotes a shift toward an anti‐inflammatory, tissue‐repairing M2 phenotype. Consistent with the gene expression results, cytokine secretion measured by ELISA demonstrated a significant increase in IL‐10 and a concomitant reduction in TNF‐α levels upon Ti/Zn treatment (Figure 5G,H). Flow cytometric analysis of CD86, a canonical M1 surface marker, further confirmed that Ti alone enhanced M1 polarization, while the Ti/Zn composite markedly attenuated CD86 expression, indicating suppression of the pro‐inflammatory phenotype (Figure 5I,J). Additionally, dual immunofluorescence staining for IL‐10 and TNF‐α visualized the spatial distribution of macrophage polarization (Figure 5K,L). Ti/Zn‐treated cells displayed enhanced IL‐10 signals and reduced TNF‐α signals, providing direct evidence of the composite's capacity to modulate macrophage behavior toward an anti‐inflammatory state.

**FIGURE 5:**
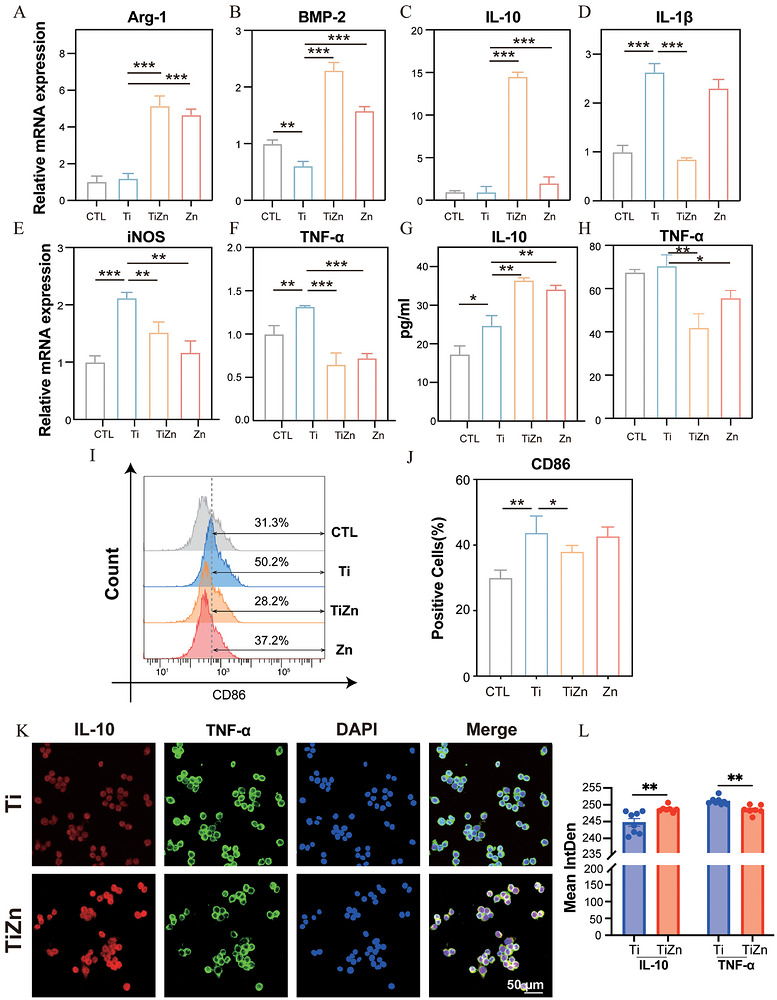
Ti/Zn composite induced M2 macrophage polarization. Ti/Zn represents the Ti/Zn‐1500 composite, which is a Zn‐infiltrated Ti scaffold with a pore size of 1500 µm. (A–F) The relative mRNA expressions of Arg‐1 (A), BMP‐2 (B), IL‐10 (C), IL‐1β (D), iNOS (E), TNF‐α (F) in macrophage after cultured in extract. (G, H) The secretion of IL‐10 (G) and TNF‐α (H) from macrophage after cultured in extract. (*n* = 3, * represents *p *< 0.05, ** represents *p* < 0.01, *t*‐test). (I) Flow cytometry analysis of CD86 in macrophage. (J) Quantification analysis of (I). (K) Dual immunofluorescence images of IL‐10 (red) and TNF‐α (green) in macrophage. (L) Quantification analysis of (K). (*n* = 10, ** represents *p* < 0.01, *t*‐test).

### 3.7 Ti/Zn Composite Induces Macrophage M2 Polarization by ERK/MAPK Signaling

To elucidate the molecular mechanisms underlying the Ti/Zn composite‐induced M2 macrophage polarization, a comprehensive transcriptomic analysis was performed on RAW264.7 macrophages cultured on different substrates. Principal component analysis (PCA) demonstrated that macrophages in the control and Ti groups clustered closely, whereas those in the Ti/Zn group showed a distinct gene expression pattern, indicating a profound transcriptomic shift triggered by zinc incorporation (Figure 6A). Differential expression analysis further confirmed substantial gene‐level changes between the Ti and Ti/Zn groups (Figure 6C). To identify the signaling pathways potentially responsible for this phenotypic transition, Kyoto Encyclopedia of Genes and Genomes (KEGG) enrichment analysis was conducted (Figure 6B). Among the top enriched pathways, the MAPK signaling cascade (mmu04010) exhibited the largest number of differentially expressed genes, suggesting that MAPK activation may play a central role in mediating the macrophage response to the Ti/Zn composite. Consistently, Gene Ontology (GO) enrichment revealed significant aggregation of genes associated with the positive regulation of immune responses (Figure 6D), in alignment with the observed shift toward an anti‐inflammatory M2 phenotype. Given that zinc homeostasis is tightly regulated by zinc transporters, the expression profiles of zinc transporter families were further examined. Notably, Scl30a1 and Scl30a3 displayed the most prominent expression differences between Ti and Ti/Zn groups (Figure 6E). This suggested that Zn^2+^ mobilized by the composite may serve as a key upstream signal. To functionally validate the involvement of Zn^2+^ and MAPK signaling in macrophage polarization, TPEN (a high‐affinity Zn^2+^ chelator) and PD98059 (a selective MEK inhibitor) were employed. Western blot analyses showed that the Ti/Zn composite selectively activated ERK, while exerting minimal effects on p38 and JNK. Importantly, ERK activation was blunted by both TPEN and PD98059, demonstrating that Zn^2+^ availability is required for ERK phosphorylation and that activation proceeds through a canonical MEK‐ERK pathway (Figure 6F,G). Cytokine analysis further revealed that inhibition of Zn^2+^ or ERK signaling diminished IL‐10 secretion while restoring TNF‐α expression, ultimately suppressing the Ti/Zn‐induced M2 polarization phenotype (Figure 6I, Figure S4).

**FIGURE 6:**
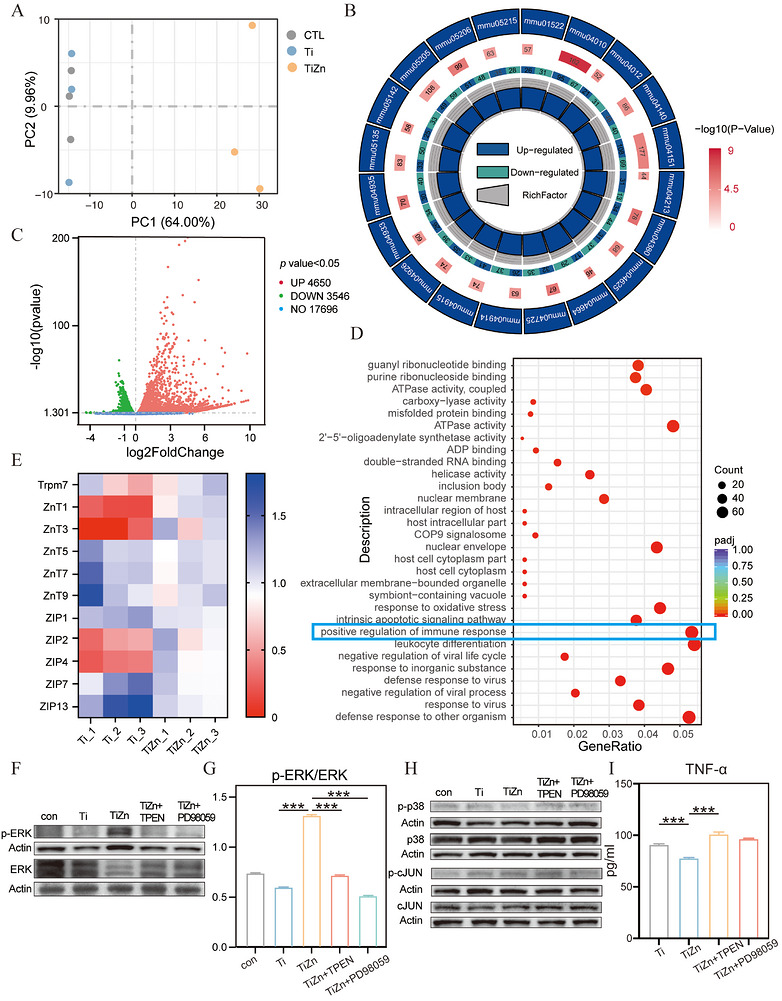
RNA‐seq and mechanistic analyses showing Ti/Zn‐induced M2 polarization via ERK/MAPK signaling. Ti/Zn represents the Ti/Zn‐1500 composite, which is a Zn‐infiltrated Ti scaffold with a pore size of 1500 µm. (A) Principle component analysis of all genes in macrophage cultured in Ti/Zn composite extract versus Ti extract. (B) Enriched KEGG pathways of Ti/Zn composite versus Ti extract. (C) Volcano plot of transcriptomic analysis of differentially expressed genes. (D) GO analysis of Ti/Zn composite versus Ti extract. (E) Heatmap analysis of differential expression genes of Zn receptors. (*n* = 3). (F) The immunoblots show the p‐ERK and ERK in macrophage after cultured in extract with or without TPEN or PD98059 administration. (G) Quantitative analyses of the immunoblots in (F). (H) The immunoblots show the p‐p38, p38, p‐cJUN, and cJUN in macrophage after cultured in extract with or without TPEN or PD98059 administration. (I) The secretion of TNF‐α from macrophage after cultured in extract with or without TPEN or PD98059 administration. (*n* = 3, ***represents *p* < 0.001, *t*‐test).

In conclusion, these findings indicate that the Ti/Zn composite modulates intracellular zinc dynamics through zinc transporters, subsequently activating the ERK signaling cascade. This Zn^2+^‐dependent ERK phosphorylation drives a transcriptional program that strengthens anti‐inflammatory pathways while attenuating pro‐inflammatory gene expression, thereby promoting macrophage polarization toward the M2 phenotype.

### 3.8 Ti/Zn Composite Restricts Mitochondrial Ca^2+^ Influx to Preserve Mitochondrial Homeostasis and Reduce Oxidative Stress

RNA sequencing analysis revealed that the Ti/Zn composite exerted pronounced regulatory effects on mitochondrial function, as reflected by significant alterations in genes associated with mitochondrial membrane transport, calcium ion homeostasis, and mitochondrial apoptosis pathways (Figure 7A). These transcriptomic changes prompted further metabolomic profiling to characterize mitochondrial metabolic remodeling induced by the Ti/Zn composite (Figure 7B). Metabolomic analysis showed that several key intermediates of the tricarboxylic acid (TCA) cycle, including succinate, L‐malate, fumarate, and 2‐oxoglutarate, were significantly downregulated, indicating a reprogramming of mitochondrial energy metabolism. In parallel, metabolites involved in fatty acid oxidation, such as propionylcarnitine, isovalerylcarnitine, and valeroylcarnitine, were markedly altered, suggesting modulation of mitochondrial substrate utilization. Notably, metabolites associated with oxidative stress regulation and antioxidant defense, including oxidized glutathione, coenzyme Q10, and plumbagin, were significantly upregulated, indicating enhanced mitochondrial antioxidant capacity in response to the Ti/Zn composite. To further investigate mitochondrial calcium regulation, calcium‐related metabolites were analyzed (Figure 7C). The results show an overall downregulation of metabolites associated with calcium ion homeostasis in the TiZn group compared with the Ti group. Collectively, these findings suggest that the TiZn composite modulates calcium‐related metabolic pathways and contributes to mitochondrial functional remodeling. Gene Ontology (GO) enrichment analysis of mitochondrial‐associated metabolites identified mitochondrial transport functions as among the most significantly affected biological processes (Figure 7D), highlighting mitochondrial transmembrane trafficking as a central component of the Ti/Zn‐mediated response. Integrated transcriptomic analysis further demonstrated that mitochondrial function was tightly regulated by zinc ions released from the Ti/Zn composite (Figure 7E, Figure S5). Specifically, genes encoding mitochondrial inner membrane protein complexes, which are essential for maintaining mitochondrial structural integrity and energy metabolism, were significantly modulated (Figure 7F). Concurrent upregulation of genes involved in mitochondrial transmembrane transport suggests enhanced and controlled exchange of ions and metabolites across the mitochondrial inner membrane, consistent with preserved mitochondrial homeostasis and reduced mitochondrial stress. In addition, the Ti/Zn composite markedly suppressed the mitochondrial release of cytochrome c, a key initiator of the intrinsic apoptotic pathway and a downstream event of mitochondrial Ca^2+^ dysregulation, indicating stabilization of mitochondrial membrane integrity. This observation was further validated at the protein level by Western blot analysis, which showed that Ti/Zn treatment significantly increased the expression of the anti‐apoptotic protein Bcl‐2 while concomitantly reducing cytochrome c expression (Figure 7G,H), confirming inhibition of Ca^2+^ dysregulation‐mediated mitochondrial apoptosis. To directly assess mitochondrial calcium homeostasis, intracellular calcium distribution was examined. Fluorescence imaging revealed that exposure to the Ti/Zn composite significantly increased cytoplasmic Ca^2+^ levels (Figure 8A) while simultaneously reducing mitochondrial Ca^2+^ accumulation (Figure 8B), indicating a redistribution of intracellular calcium ions. To evaluate the functional consequences of this redistribution, mitochondrial membrane potential (ΔΨm) was assessed using JC‐1 staining. Cells treated with the Ti/Zn composite exhibited a significantly higher red/green fluorescence ratio (Figure 8C,D), reflecting improved mitochondrial membrane stability and preserved mitochondrial function. These in vitro findings were further corroborated in vivo. Immunofluorescence analysis of peri‐implant tissues demonstrated a marked reduction in oxidative stress levels surrounding Ti/Zn implants compared with controls (Figure 8E,F), confirming that the Ti/Zn composite effectively alleviates mitochondrial‐associated oxidative stress within the implant microenvironment.

**FIGURE 7:**
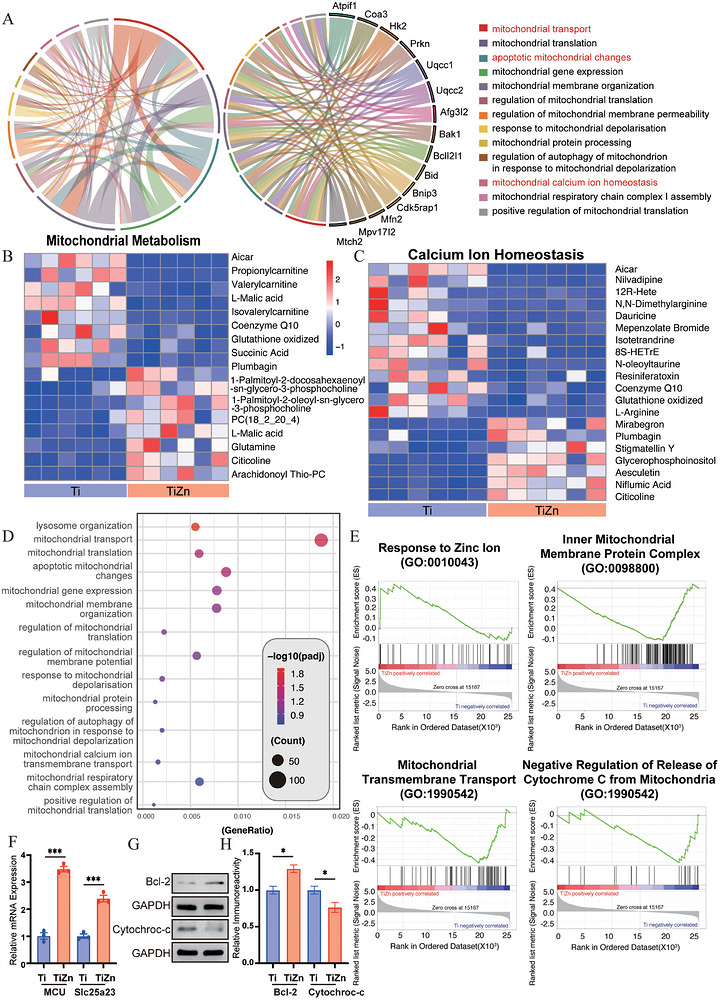
Ti/Zn composite maintains mitochondrial stability through regulating Ca^2+^ flow: RNA‐seq and metabolomics evidence. Ti/Zn represents the Ti/Zn‐1500 composite, which is a Zn‐infiltrated Ti scaffold with a pore size of 1500 µm. (A) Chord diagram of GO‐enriched mitochondrial genes regulated by the Ti/Zn composite. (B) Heatmap of metabolites involved in mitochondrial metabolic pathways regulated by the Ti/Zn composite. (C) Heatmap of metabolites involved in calcium ion homeostasis pathways regulated by the Ti/Zn composite. (D) KEGG pathway enrichment of the metabolome. (E) GSEA of GO pathway enrichment based on RNA‐seq analysis. (F) The relative mRNA expressions of MCU and Slc25a23. (*n* = 3, ***represents *p* < 0.001, *t*‐test). (G) The immunoblots show the Bcl‐2 and Cytochroc‐c in macrophage after cultured in extract. (H) Quantitative analyses of the immunoblots in (G). (*n* = 3, * represents *p* < 0.05, *t*‐test).

**FIGURE 8:**
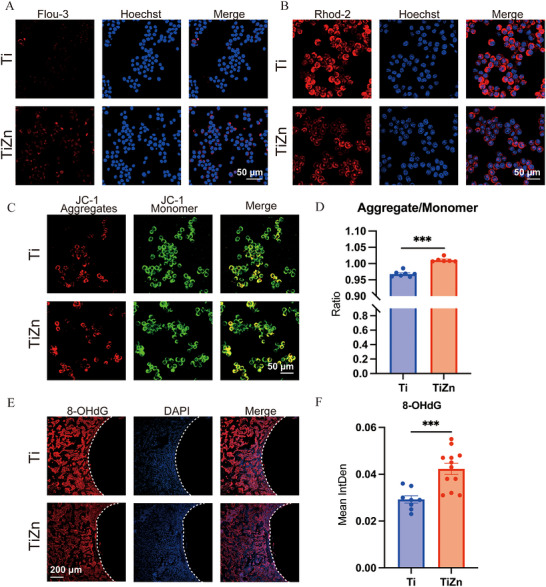
Ti/Zn composite suppresses Ca^2+^ influx from the cytoplasm into mitochondria and alleviates oxidative stress. Ti/Zn represents the Ti/Zn‐1500 composite, which is a Zn‐infiltrated Ti scaffold with a pore size of 1500 µm. (A) Fluorescence imaging of cytoplasmic Ca^2+^ levels in macrophages (Fluo‐3 staining). (B) Fluorescence imaging of mitochondrial Ca^2+^ levels in macrophages (Rhod‐2 staining). (C) Fluorescence imaging of mitochondrial membrane potential (ΔΨm) in macrophages (JC‐1 staining). (D) Quantification of fluorescence intensity in (C). (E) Fluorescence imaging of oxidative stress in peri‐implant tissue (8‐OHdG staining). (F) Quantification of fluorescence intensity in (E). (*n* = 10, ***represents *p* < 0.001, *t*‐test).

Taken together, these results demonstrate that the Ti/Zn composite restricts Ca^2+^ influx from the cytoplasm into mitochondria, thereby preserving mitochondrial homeostasis and suppressing oxidative stress.

### 3.9 The Ti/Zn Composite Promotes Bone Tissue Regeneration through the Action of Macrophages

To further clarify whether immunoregulatory effects induced by the Ti/Zn composite contribute to osteogenesis, an indirect co‐culture system was established in which MC3T3‐E1 pre‐osteoblasts were cultured in conditioned medium collected from macrophages previously exposed to Ti/Zn extract (Figure 9A). MC3T3‐E1 cells cultured in Ti/Zn‐conditioned medium (Ti/ZnCM) exhibited a significant increase in cell viability at all time points, as demonstrated by CCK‐8 assays (Figure 9B). Early‐stage osteogenic differentiation was markedly enhanced in the Ti/ZnCM group, evidenced by stronger ALP staining and significantly higher ALP enzymatic activity compared to the control and TiCM groups (Figure 9C,D). Consistent with this, RT‐qPCR analysis revealed substantial upregulation of osteogenic genes—including COL1, ALP, RUNX‐2, and OSX after 3 days of Ti/ZnCM stimulation (Figure 9E–H). At the protein level, immunofluorescence staining confirmed enhanced expression of COL1, RUNX‐2, and ALP in MC3T3‐E1 cells cultured in Ti/ZnCM for 7 days (Figure 9I–L). These results indicate that macrophages exposed to the Ti/Zn composite secrete a pro‐osteogenic cytokine profile capable of driving both early and mid‐stage osteogenic differentiation. Morphologically, MC3T3‐E1 cells maintained their characteristic fibroblast‐like phenotype in all groups. However, Ti/ZnCM and ZnCM markedly improved cell spreading and increased the cell‐covered area at both 4 and 24 h (Figure 9M). In addition, Ti/ZnCM increased the number of mineralized nodules (Figure 9N). These findings suggest enhanced cytoskeletal organization and adherence‐features typically associated with favorable osteogenic microenvironments. Importantly, these in vitro findings were consistent with in vivo observations. IHC staining of peri‐implant tissue demonstrated increased expression of OCN and RUNX‐2 around Ti/Zn implants compared with other groups (Figure 9O,P), further confirming the ability of the Ti/Zn composite to promote osteogenesis by modulating immune‐cell behavior at the implant interface.

FIGURE 9Reciprocal interactions between osteoinductive and immunomodulatory characteristics of Ti/Zn composite. CTL: control group, CTLCM: control medium group, TiCM: Ti extract cultured macrophages condition medium, Ti/ZnCM: Ti/Zn‐1500 extract cultured macrophages condition medium, ZnCM: Zn extract cultured macrophages condition medium. (A) Schematic representation of the experimental design. (B) The cell viability of MC3T3‐E1 cells after cultured with condition medium from macrophages for 1 day, 2 days, and 3 days. (*n* = 3, * represents *p* < 0.05, ** represents *p* < 0.01, ***represents *p* < 0.001, one‐way ANOVA). (C) Quantitative analysis of ALP activity in MC3T3‐E1 cells after cultured with condition medium from macrophages for 7 days. (*n* = 3, ** represents *p* < 0.01, ***represents *p* < 0.001, *t*‐test). (D) The ALP staining of MC3T3‐E1 cells after cultured with condition medium from macrophages for 3 days and 7days. (E–G) The relative mRNA expressions of COL1 (E), ALP (F), RUNX2 (G), and OSX (H) in MC3T3‐E1 cells after cultured with condition medium from macrophages for 3 days. (*n* = 3, * represents *p* < 0.05, ** represents *p* < 0.01, ***represents *p* < 0.001, *t*‐test). (I) The immunoblots show the COL1, RUNX2, and ALP in MC3T3‐E1 cells after cultured with condition medium from macrophages for 7 days. (I–L) Quantitative analyses of the immunoblots in (H). (*n* = 3, * represents *p* < 0.05, ** represents *p* < 0.01, ***represents *p* < 0.001, *t*‐test). (M) Phalloidin staining showing F‐actin distribution in MC3T3‐E1 cells. (N) ARS staining of MC3T3‐E1 cells cultured with condition medium from macrophages for 28 days. (O, P) Representative immunohistochemical images of OCN (O) and RUNX2 (P) in peri‐implant tissue.

**Figure:**
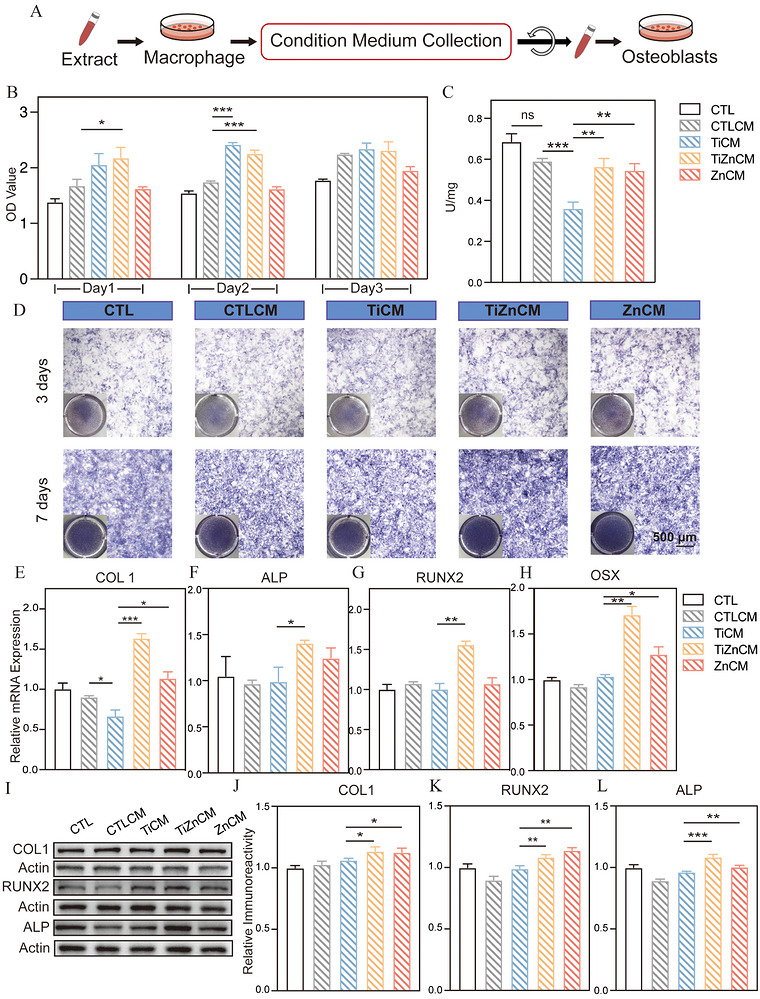

**Figure:**
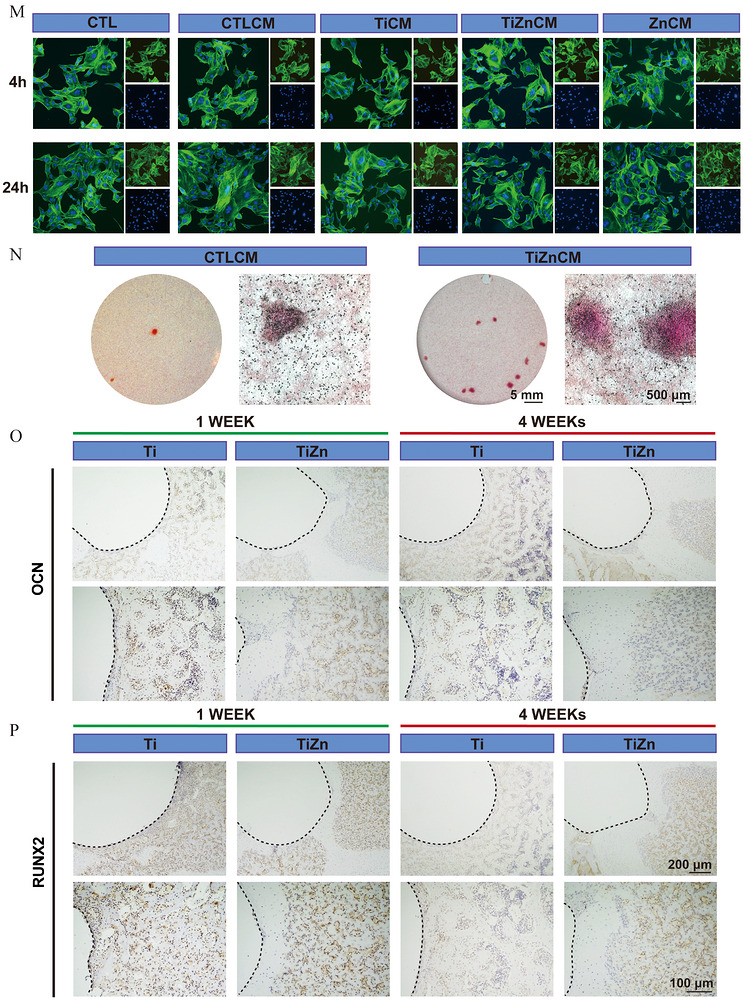

In conclusion, these results provide compelling evidence that the Ti/Zn composite triggers macrophage M2‐biased polarization and remodels the secretory phenotype toward an anti‐inflammatory, pro‐regenerative profile. The resulting cytokine milieu enhances osteoblast proliferation, differentiation, and matrix protein synthesis, highlighting a macrophage‐mediated immuno‐osteogenic mechanism underlying the superior bone‐forming capacity of the Ti/Zn composite.

## 4 Discussion

In this study, we designed and fabricated two 3D‐printed porous titanium scaffolds with pore diameters of 1200 µm and 1500 µm, into which molten zinc was infiltrated to construct Ti/Zn‐1200 and Ti/Zn‐1500 composites. This design strategy aimed to integrate the mechanical robustness of titanium with the immunomodulatory bioactivity of zinc, while enabling controlled degradation and ion release. Comparative evaluation of degradation behavior revealed that the Ti/Zn‐1500 composite exhibited a more favorable degradation profile, characterized by an appropriate degradation rate, sustained Zn^2+^ release, and superior mechanical performance. Nanoindentation analysis further demonstrated that, compared with Ti/Zn‐1200, the Ti/Zn‐1500 composite possessed biomechanical properties that more closely matched those of native bone tissue. Based on these results, Ti/Zn‐1500 was selected for subsequent biological performance evaluations. We then systematically investigated the osteogenic effects of the Ti/Zn composite with particular emphasis on its ability to modulate macrophage behavior and reshape the peri‐implant immune microenvironment. To elucidate the immunoregulatory mechanisms, macrophages were exposed to diluted Ti/Zn composite extracts, with extract concentrations determined according to cytotoxicity assessments following ISO 10993 standards. Notably, the Zn^2+^ concentration in the extract at the maximum safe level was comparable to that observed in simulated body fluid, indicating favorable biocompatibility and physiological relevance. Using this platform, we demonstrated that the Ti/Zn composite promotes osteogenesis through immune modulation, primarily by regulating macrophage polarization and downstream biological responses. The detailed cellular and molecular mechanisms underlying this immuno‐osteogenic coupling are summarized schematically in Figure 10. A central finding of this study is that the osteogenic advantage of the Ti/Zn composite is closely linked to its ability to actively regulate macrophage behavior through zinc‐mediated intracellular signaling rather than acting as a passive osteoconductive scaffold. Our data collectively indicate that Zn^2+^ released from the Ti/Zn composite functions as a key bioactive signal that reshapes macrophage intracellular calcium distribution, thereby triggering downstream signaling events that determine inflammatory phenotype and cellular metabolic status. Specifically, the Ti/Zn composite increased cytoplasmic Ca^2+^ levels while restricting excessive Ca^2+^ influx into mitochondria, preventing mitochondrial Ca^2+^ overload. This redistribution of intracellular calcium is particularly significant, as mitochondrial Ca^2+^ dysregulation is known to impair oxidative phosphorylation, promote cytochrome c release, and trigger oxidative stress‐associated apoptosis [36]. By maintaining mitochondrial Ca^2+^ homeostasis, the Ti/Zn composite preserved mitochondrial membrane potential, stabilized mitochondrial inner membrane protein complexes, and suppressed activation of the intrinsic apoptotic pathway, as evidenced by increased Bcl‐2 expression and reduced cytochrome c release. At the signaling level, transcriptomic and pharmacological analyses demonstrated that Zn^2+^‐dependent calcium regulation selectively activated the ERK/MAPK pathway, rather than the p38 or JNK branches, positioning ERK as a key molecular node linking ionic cues to macrophage phenotype determination. The ERK‐driven maintenance of mitochondrial function and reduction of oxidative stress likely provides the metabolic foundation required for sustained M2 macrophage polarization. Functionally, this mitochondrial stabilization and anti‐oxidative shift translated into a macrophage secretory profile dominated by anti‐inflammatory and pro‐regenerative mediators, which in turn enhanced osteogenic differentiation of surrounding osteoprogenitor cells. The indirect co‐culture experiments confirmed that macrophage‐conditioned media derived from Ti/Zn exposure significantly promoted osteoblast proliferation, differentiation, and matrix protein expression, establishing a clear immuno‐osteogenic coupling mechanism.

**FIGURE 10:**
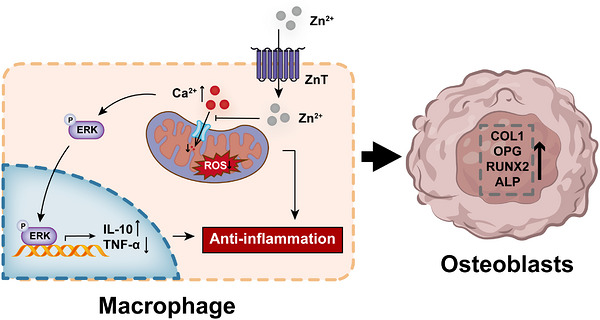
Schematic illustration of the cellular and molecular mechanisms underlying immuno‐osteogenic coupling induced by the Ti/Zn composite. Zinc ions released from the Ti/Zn composite enter macrophages through ZnT transporters and subsequently regulate mitochondrial membrane receptors, thereby limiting the influx of intracellular Ca^2+^ into mitochondria. This redistribution of calcium exerts dual regulatory effects: cytoplasmic Ca^2+^ accumulation activates the ERK/MAPK signaling pathway, promoting the anti‐inflammatory polarization of macrophages, while reduced mitochondrial Ca^2+^ levels suppress mitochondrial oxidative stress and preserve mitochondrial homeostasis. Together, these coordinated effects drive macrophages toward an anti‐inflammatory, pro‐regenerative phenotype, which in turn enhances osteogenic activity and establishes an immuno‐osteogenic microenvironment mediated by the Ti/Zn composite.

Taken together, these findings suggest that the Ti/Zn composite operates through an ion‐driven immunometabolic mechanism, in which Zn^2+^‐regulated Ca^2+^ flux orchestrates ERK/MAPK signaling and mitochondrial homeostasis in macrophages. This coordinated regulation suppresses oxidative stress and inflammatory signaling while fostering a regenerative immune microenvironment that ultimately supports robust bone formation.

Additive manufacturing techniques for metals, particularly three‐dimensional (3D) printing using selective laser or electron beam melting, offer a powerful approach to processing metallic materials. Interpenetrating Phase Composites (IPCs), developed through additive manufacturing techniques, address the limitations of individual materials and enhance overall performance by synergistically combining the distinct properties of two materials [37, 38]. Several studies have developed interpenetrating phase composites by infiltrating metals into Ti‐based scaffolds, demonstrating improvements in the toughness and fatigue strength of implants while also providing enhanced biological properties [34, 39, 40, 41, 42]. In addition, for partially degradable composites, the degraded metal creates space for the growth of new bone, while the new bone provides mechanical support in the initial stage. Among degradable metals, Mg, Fe, and Zn are currently considered the leading options. Fe exhibits a relatively slow degradation rate, with insoluble degradation products persisting in physiological environments, and its high mechanical strength can lead to stress shielding [43]. In contrast, the rapid degradation rate and hydrogen release remain key concerns limiting the application of Mg. Dou et al. investigated the infiltration of Mg into Ti scaffolds. The degradation of Mg generates hydrogen gas, and the accelerated degradation rate alters the pH value of the surrounding tissue [44]. Zinc exhibits more favorable corrosion behavior under physiological conditions compared to other biodegradable metals, such as magnesium and iron. The corrosion mechanism of Zn is regulated by the following reactions [2]:

$$\mathrm{Anodic}\;\mathrm{reaction}:\to \mathrm{Zn}\to Zn^{2+}+2e^{-}$$

$$\mathrm{Cathodic}\;\mathrm{reaction}:\to 2H_{2}O+O^{2}+4e^{-}\to 4OH^{-}$$

$$\mathrm{Overall}:\to Zn^{2+}+2OH^{-}\to \mathrm{Zn}{\left(\mathrm{OH}\right)}_{2}$$

$$\mathrm{Other}\;\mathrm{reactions}:\to \mathrm{Zn}{\left(\mathrm{OH}\right)}_{2}\to \mathrm{ZnO}+H_{2}O$$

$$6\mathrm{Zn}{\left(\mathrm{OH}\right)}_{2}+Zn^{2+}+2Cl^{-}\to 6\mathrm{Zn}{\left(\mathrm{OH}\right)}_{2}\cdot \mathrm{ZnC}l_{2}$$

$$Zn^{2+}+2\mathrm{HP}{O_{4}}^{-2}+2OH^{-}+2H_{2}O\to Zn_{3}{\left(PO_{4}\right)}_{2}\cdot 4\left(H_{2}O\right)$$

Yu et al. investigated the infiltration of Zn into Ti6Al4V scaffolds. Zn demonstrates superior deformation capabilities compared to Ti6Al4V, facilitating smoother stress transfer between materials. Under upper‐stress conditions, it promotes the gradual transfer of stress from the upper layer to the lower layer, effectively resisting crack propagation and ensuring uniform deformation, thereby enhancing the composite's toughness and energy absorption capacity [42]. In this study, nanoindentation testing was performed to evaluate the mechanical properties of the Ti/Zn composites and to monitor their evolution during immersion. The distinct nanoindentation trends between Ti/Zn‐1200 and Ti/Zn‐1500 highlight the critical role of composite microstructure in governing biomechanical durability during degradation (Figure 2). The rapid early displacement and hardness reduction of the Zn phase in Ti/Zn‐1200 indicate an accelerated corrosion regime, likely driven by a more reactive Ti–Zn interfacial coupling and a less compact Zn infiltration state. In contrast, the Ti/Zn‐1500 composite maintained comparatively stable displacement and a moderate, gradual hardness decline, suggesting a denser and more mechanically resilient architecture that mitigates excessive electrochemical corrosion. Importantly, the Ti‐phase hardness (∼5 GPa) in Ti/Zn‐1500 closely approximates that of native bone, a feature that is advantageous for load transfer and for minimizing stress‐shielding at the bone–implant interface. These findings collectively support the selection of Ti/Zn‐1500 as a mechanically optimized, bioactive composite capable of balancing structural integrity with controlled Zn^2+^ ‐mediated biodegradation for bone implant applications.

We designed a cathodic protection strategy employing zinc as a sacrificial anode to mitigate titanium degradation. It can be observed that the concentration of released Zn^2+^ and the corrosion rate in the Ti/Zn group are slightly higher compared to those in the pure zinc group (Figure 1D). Although the extract of the Ti/Zn composite shows cytotoxicity in cell experiments (Figure S6), in vivo studies have demonstrated not only the satisfactory biocompatibility of the Ti/Zn composite but also its ability to promote osteogenesis (Figure 3). This result is consistent with a wide range of previous studies. A series of in vitro studies have demonstrated that the cytotoxicity of biodegradable Zn and its alloys primarily depends on the type of Zn‐based material [45], the cell lines used [46], and the experimental conditions [47, 48]. Processing technologies and alloying elements can influence the toxicity of Zn‐based biomaterials by modifying their microstructure and corrosion characteristics [49]. The corrosion behavior of metals is influenced by metallurgical factors such as alloy composition, phase precipitation, and the segregation of alloying elements and impurities [50]. In addition, surface treatments play a significant role in determining corrosion behavior [51, 52]. The composition of the pre‐cultivation medium and the surrounding environment of the biomaterial are also critical factors [47, 48, 53]. Therefore, the disparity between in vivo and in vitro environments may result in markedly different corrosion rates, potentially explaining the variability in cytotoxicity results. The different corrosion rate between in vitro and in vivo studies was certified by other studies. In a study of Zn‐Li‐Mn alloy designed for gastrointestinal anastomosis, the alloys were immersed in simulated gastric fluid for 28 days, and the weight loss ratio in SGF 25.8%. Furthermore, the alloy was also utilized for gastrointestinal anastomosis in pig model, and its remaining volume was 89 ± 2% after 12 weeks [54]. This result suggest the in vitro degradation is much higher than in vivo degradation. In contrast to this study, another study of Zn‐1.0Cu‐0.5‐Ca designed for subcuticular absorbable staples suggest the in vivo degradation (198 ± 54, 112 ± 28, and 70 ± 24 µm/y after 1, 4, and 12 weeks) is much higher than the in vitro degradation(69 ± 8, 53 ± 5, and 21 ± 4 µm/y after 1, 4, and 12 weeks) [55]. Current in vitro evaluation standards (ISO‐10993) for bio materials have limitations in predicting in vivo acceptance [56]. It is suggested to dilute the standard extract for in vitro bio compatibility testing in the situation of biodegradable metallic materials [57]. Therefore, in this experiment, the concentration of Zn^2+^ in the extract prepared according to ISO‐10993 and the concentration of Zn^2+^ in the medium replaced daily, simulating body fluid circulation, were measured. The results indicated that the concentration of Zn^2+^ released by the Ti/Zn composite in the daily‐replaced medium remained relatively stable over time, and the Zn^2+^ concentration in the medium was comparable to that in the standard extract diluted five times (Figure 1G,H). Besides the different corrosion rate, the different cytotoxicity in in vivo and in vitro study may due to the different response of cells to the released Zn^2+^. In contrast to other metals (such as iron, copper, and mercury) that can accumulate excessively in the organism, leading to toxic levels, the uptake, subcellular distribution, storage, and excretion of Zn are efficiently regulated. Key components in maintaining Zn homeostasis include the Zn importers Zrt‐ and Irt‐like proteins (ZIP) and Zn transporters (ZnT), which manage Zn fluxes across the plasma membrane and within cellular compartments [58, 59]. Therefore, the mRNA expression of ZIPs and ZnTs was first measured in this study (Figure 6E). The results indicated in the ZIP family, which increases cytosolic Zn levels by allowing Zn uptake from the extracellular environment or efflux from intracellular organelles to the cytoplasm, Slc39a2 (ZIP2) and Slc39a4 (ZIP4) were slightly increased. In the ZnT family, which carries Zn out of the cell or into intracellular storage compartments, Slc30a1 (ZnT1) and Slc30a3 (ZnT3) significantly increased. In addition to intracellular Zn^2+^, the presence of Zn^2+^ in extracellular transport vehicles is a more recent discovery. Zn^2+^ has been detected in presynaptic vesicles, which are released upon exocytic stimulation, indicating Zn^2+^ as a potential intercellular signaling molecule [60]. This phenomenon may also exist in secretory cells beyond neurons. Consequently, the Zn^2+^ released by the Ti/Zn composite could exist both within cells and in transporter vehicles, potentially mitigating cellular toxicity.

Zinc is the second most prevalent trace metal in the human body. Zn^2+^ have been widely reported to exert a dose‐dependent and biphasic regulatory effect on macrophage polarization. Moderate Zn^2+^ levels, typically in the micromolar range, have been shown to promote M2 polarization and anti‐inflammatory responses, potentially through inhibition of NF‐κB signaling and modulation of oxidative stress pathways [61]. In contrast, insufficient Zn availability may impair macrophage function, whereas excessive Zn^2+^ exposure can induce cytotoxicity and disrupt redox homeostasis, thereby attenuating its immunoregulatory benefits [62, 63]. Notably, Zn‐containing biomaterials releasing Zn^2+^ within a physiologically relevant range have demonstrated enhanced M2 polarization and tissue regeneration outcomes [14]. In the present study, however, Zn^2+^ exposure is not administered as predefined concentrations but is dynamically generated through ion release from the Ti/Zn composite. Consequently, the local Zn^2+^ microenvironment at the cell–material interface is time‐dependent and strongly influenced by scaffold architecture, including pore structure and Ti/Zn composition, rather than representing a static concentration gradient. This material‐driven release behavior may better mimic in vivo ion exposure conditions and provides a more physiologically relevant context for macrophage regulation. Therefore, the observed promotion of M2 polarization is likely attributed to a controlled Zn^2+^ microenvironment established by the composite rather than discrete external dosing concentrations.

Changing phosphorylation status predominantly regulates signal transduction by activating or inactivating existing signaling molecules post‐translationally. In this study, the detection of ERK phosphorylation indicated the activation of the MAPK/ERK signaling pathway (Figure 6F,G). Treatment with the ERK inhibitor PD98059 reversed macrophage polarization from the M2 to the M1 phenotype, suggesting that MAPK/ERK signaling activation plays a role in the Ti/Zn composite‐induced promotion of M2 macrophage polarization. Furthermore, the administration of TPEN, a zinc chelator, suppressed the activation of the MAPK/ERK signaling pathway and similarly reversed macrophage polarization from M2 to M1, indicating that Zn^2+^ transporters are involved in modulating macrophage polarization (Figure 6F–I). Multiple Slc30/Slc39 members are expressed in macrophage. These transporters play an essential role in macrophage under both physiological and pathological conditions. Modulating the expression of these transporters can influence the functional behavior of macrophages [64]. In this study, the gene expression of Slc30/Slc39 members was initially assessed and subsequently validated the protein expression of receptors associated with upregulated gene expression. The findings indicate that Slc30a3 expression was explicitly enhanced by the Ti/Zn composite.

Studies have indicated that zinc flux is associated with the inhibition of MAPK phosphatases in neurons, though this connection has yet to be demonstrated in immune cells [65]. Sindreu et al. showed that the absence of ZnT3 reduces ERK activation in hippocampus mossy fiber terminals [66]. Consistent with this finding, knocking down ZnT3 in SH‐SY5Y cells also significantly suppressed endoplasmic reticulum stress‐induced ERK1/2 phosphorylation [67]. In this study, our sequence results suggest the Ti/Zn composite has significant effects on some signaling pathways (Figure 6A–D). Among them, the MAPK signaling pathway genes exhibit more significant changes compared to genes in other signaling pathways. The underlying mechanism of the Zn effect on macrophage polarization is controversial. Research has demonstrated that the TLR4‐NF‐κB [68] and TLR6‐MAPK [69] signaling pathways are upregulated in the early stages, primarily influencing M1 macrophages, while in the later stages, the CaSR‐PI3K‐Akt1 signaling pathway becomes more prominent [70]. Yamasaki et al. reported zinc is able to activate both ERK and cJUN in MAPK signaling [71]. However, in this study, only ERK was activated by the Ti/Zn composite (Figure 6F,G). The discrepancies between studies may be attributed to differences in zinc concentration, alterations in zinc status, and the distinct activation patterns of zinc in various cell types.

Mitochondria, as the central organelles responsible for energy production in eukaryotic cells, play a crucial role in regulating macrophage functions through energy metabolism and oxidative stress control. They also participate in various signaling pathways by continuously and dynamically interacting with their cellular environment. In the context of biomaterials, implanted materials drastically alter the local tissue milieu; therefore, elucidating mitochondria‐related signaling responses has great significance. Mitochondrial activity is tightly regulated by ion‐dependent communication between intracellular compartments, with Ca^2+^ serving as a key second messenger. In this study, both transcriptomic and metabolomic analyses indicated that the Ti/Zn composite could regulate intracellular calcium homeostasis. The transcriptomic results further suggested a potential effect on mitochondrial membrane channel proteins. However, we found that the Ti/Zn composite primarily affected the gene transcription levels of these channel proteins, while no significant change was observed at the protein level (Figure 7A–F, Figure S7). This discrepancy may be attributed to post‐transcriptional regulatory mechanisms. The phosphorylation status of mitochondrial calcium transport‐related proteins was not evaluated in the present study. Due to the limited availability of well‐validated phosphorylation‐specific antibodies and technical challenges associated with detecting these modifications, further investigation will be required to clarify the role of phosphorylation‐mediated regulation in Zn^2+^‐associated mitochondrial signaling. We further monitored Ca^2+^ levels in both the cytoplasm and mitochondria, demonstrating that the Ti/Zn composite suppresses mitochondrial Ca^2+^ influx from the cytoplasm. Previous studies have shown that Zn^2+^ can reduce mitochondrial Ca^2+^ accumulation by competing for calcium transporters, a finding consistent with our RNA sequencing results. Billur et al. also reported that excessive cytoplasmic Zn^2+^‐induced Ca^2+^ elevation is accompanied by an increase in lysosome number [72]. Accordingly, we evaluated lysosomal stability using immunostaining, which revealed no appreciable difference between the Ti and Ti/Zn groups, indicating that the composite modulates calcium signaling without lysosomal disruption (Figure S8).

Exploring zinc metabolism and intracellular zinc signaling pathways is critical for the rational design of zinc‐containing implants. The Zn infiltration process is intrinsically constrained within a narrow processing window governed by the balance between melt fluidity, wettability, and the thermal stability of the additively manufactured Ti scaffold. Insufficient thermal input limits capillary‐driven infiltration, resulting in incomplete Zn filling, whereas excessive thermal exposure induces structural degradation of the Ti framework, particularly in the thin struts of the porous architecture [42]. Consequently, only a limited parameter range enables both effective Zn infiltration and structural integrity, inherently restricting systematic parameter variation and quantitative comparison under non‐viable conditions.

From a translational perspective, further investigation into controlled Zn^2+^ release and its interaction with the implant‐associated immune microenvironment is warranted. The integration of selective laser melting and pressureless infiltration enables precise regulation of scaffold architecture, including porosity and pore size, thereby tuning the Ti/Zn ratio. This design strategy provides a means to modulate Zn ion release kinetics and the evolution of biomechanical properties during degradation, ultimately enabling the development of Ti/Zn composites tailored for specific application scenarios.

While Zn is an essential trace element, excessive accumulation may lead to cytotoxic and systemic effects. Therefore, controlling Zn^2+^ release kinetics is critical for ensuring long‐term biosafety of Zn‐containing implants. Systematic long‐term assessments, including both in vivo and in vitro evaluations, are expected to provide complementary quantitative insights. From a materials perspective, key aspects such as corrosion kinetics, mechanical property degradation, and fatigue performance require further investigation. From a biological perspective, the effects of material‐released ions on the local tissue microenvironment and systemic responses also warrant further clarification. These aspects have now been acknowledged as limitations of the present study and will be addressed in future work.

## Author Contributions

**Zengqian Liu**: Writing – review and editing, methodology. **Yueyang Qiu**: methodology. **Bing Bai**: validation. **Qiyue Zhang**: data curation. **Haocheng Suo**: software. **Qiang Wang**: conceptualization, writing – review and editing, project administration, methodology, supervision, formal analysis, investigation. **Wanyi Huang**: writing – original draft, data curation, software, validation, visualization. **Qing Zhou**: project administration, writing – review and editing, funding acquisition, resources.

## Conflicts of Interest

The authors declare that the research was conducted in the absence of any commercial or financial relationships that could be construed as a potential conflict of interest.

## Supporting information




**Supporting File 1**: advs76941‐sup‐0001‐SuppMat.docx.

**Supporting File 2**: advs76941‐sup‐0002‐FigureS1–S8.zip.

## Data Availability

Data available on request from the authors.
