# Supplementary material for: Ion‐Driven Regulation of Ca2 + Flow by Ti/Zn Composite Activates ERK/MAPK Signaling and Maintains Mitochondrial Homeostasis to Promote Macrophage‐Mediated Bone Regeneration
Source: Adv Sci (Weinh). 2026 Aug 5;13(60):e76941. doi: 10.1002/advs.76941 (PMC13440380; doi:10.1002/advs.76941)
Supplement: Supplementary file 2 [file ADVS-13-e76941-s002.docx]

**Table 1 qPCR Primer Sequences**

|  | Forward(5’-3’) | Reverse(5’-3’) |
| --- | --- | --- |
| Actin | GTGACGTTGACATCCGTAAAGA | GCCGGACTCATCGTACTCC |
| IL-10 | CTTACTGACTGGCATGAGGATCA | GCAGCTCTAGGAGCATGTGG |
| BMP-2 | TCTTCCGGGAACAGATACAGG | TGGTGTCCAATAGTCTGGTCA |
| Arg-1 | CTCCAAGCCAAAGTCCTTAGAG | AGGAGCTGTCATTAGGGACATC |
| IL-1β | GCAACTGTTCCTGAACTCAACT | ATCTTTTGGGGTCCGTCAACT |
| iNOS | ACATCGACCCGTCCACAGTAT | CAGAGGGGTAGGCTTGTCTC |
| TNF-α | CAGGCGGTGCCTATGTCTC | CGATCACCCCGAAGTTCAGTAG |
| CD86 | TGTTTCCGTGGAGACGCAAG | TTGAGCCTTTGTAAATGGGCA |
| CD206 | CTCTGTTCAGCTATTGGACGC | CGGAATTTCTGGGATTCAGCTTC |
| MCU | GAACACGTCCCCTGCTTTGT | ACGTCATCAGAGGGCACAAC |
| Slc25a23 | TCCTCTGACTGGGATGCTGA | ATGTGACCATCTTGGTTCCG |
| COL1 | TAAGGGTCCCCAATGGTGAGA | GGGTCCCTCGACTCCTACAT |
| ALP | GCACCTGCCTTACCAACTCT | GTGGAGACGCCCATACCATC |
| RUNX2 | TTCAACGATCTGAGATTTGTGGG | GGATGAGGAATGCGCCCTA |
| OSX | TTTCTGCGGCAAGAGGTTCA | TTGCTCAAGTGGTCGCTTCT |

**Table 2 Scaffold Porosity and Zn Volume Fraction in Ti–Zn Composite**

| The pore size of Ti Scaffold | Ti Scaffold porosity | Zn proportion in TiZn composite | p value |
| --- | --- | --- | --- |
| 1200 μm | 38.52±1.8% | 37.44±2.3% | 0.548 |
| 1500 μm | 50.53±2.4% | 56.98±8.6% | 0.281 |

**Table 3 Ti-Zn Composite EDS**

| **Element** | **1200 μm** | | **1500 μm** | |
| --- | --- | --- | --- | --- |
|  | **Wt%** | **Wt% σ** | **Wt%** | **Wt% σ** |
| **Zn** | 52.06 | 0.31 | 61.58 | 0.22 |
| **O** | 32.89 | 0.24 | 35.05 | 0.21 |
| **Ti** | 4.07 | 0.06 | 2.13 | 0.06 |
| **Cl** | 0.69 | 0.04 | 0.83 | 0.05 |

**Supplementary Figure Legend**

**Supplementary Fig. 1.** Melting Curve Analysis of qPCR Primers.

**Supplementary Fig. 2.** XRD patterns of Ti/Zn-1200 composites before and after immersion for different durations.

**Supplementary Fig. 3.** H&E Staining of Major Organs in Rat Bone Defect Model.

**Supplementary Fig. 4.** The secretion of IL-10 from macrophage after cultured in extract with or without TPEN or PD98059 administration.

**Supplementary Fig. 5.** Heatmap of differentially expressed genes involved in GO enrichment analysis.

**Supplementary Fig. 6.** CCK-8 assay results for RAW 264.7 cells cultured with extracts at different dilution times.

**Supplementary Fig. 7.** Western Blot Analysis of the Mitochondrial Membrane Channel Protein MCU.

**Supplementary Fig. 8.** Fluorescence imaging of lysosome in macrophages (Lyso-Track).
